# In vivo bone regeneration assessment of offset and gradient melt electrowritten (MEW) PCL scaffolds

**DOI:** 10.1186/s40824-020-00196-1

**Published:** 2020-10-01

**Authors:** Naghmeh Abbasi, Ryan S. B. Lee, Saso Ivanovski, Robert M. Love, Stephen Hamlet

**Affiliations:** 1 School of Dentistry and Oral Health, Griffith University, Gold Coast Campus, Southport, Queensland 4215 Australia; 2 Menzies Health Institute Queensland, Griffith University, Gold Coast Campus, Southport, Queensland 4215 Australia; 3 School of Dentistry, University of Queensland, Herston Campus, Herston, Queensland 4006 Australia

**Keywords:** Pore size, Melt electrowriting, Bone tissue engineering, Angiogenesis, Scaffold, Poly (ε-caprolactone)

## Abstract

**Background:**

Biomaterial-based bone tissue engineering represents a promising solution to overcome reduced residual bone volume. It has been previously demonstrated that gradient and offset architectures of three-dimensional melt electrowritten poly-caprolactone (PCL) scaffolds could successfully direct osteoblast cells differentiation toward an osteogenic lineage, resulting in mineralization. The aim of this study was therefore to evaluate the in vivo osteoconductive capacity of PCL scaffolds with these different architectures.

**Methods:**

Five different calcium phosphate (CaP) coated melt electrowritten PCL pore sized scaffolds: 250 μm and 500 μm, 500 μm with 50% fibre offset (offset.50.50), tri layer gradient 250–500-750 μm (grad.250top) and 750–500-250 μm (grad.750top) were implanted into rodent critical-sized calvarial defects. Empty defects were used as a control. After 4 and 8 weeks of healing, the new bone was assessed by micro-computed tomography and immunohistochemistry.

**Results:**

Significantly more newly formed bone was shown in the grad.250top scaffold 8 weeks post-implantation. Histological investigation also showed that soft tissue was replaced with newly formed bone and fully covered the grad.250top scaffold. While, the bone healing did not happen completely in the 250 μm, offset.50.50 scaffolds and blank calvaria defects following 8 weeks of implantation. Immunohistochemical analysis showed the expression of osteogenic markers was present in all scaffold groups at both time points. The mineralization marker Osteocalcin was detected with the highest intensity in the grad.250top and 500 μm scaffolds. Moreover, the expression of the endothelial markers showed that robust angiogenesis was involved in the repair process.

**Conclusions:**

These results suggest that the gradient pore size structure provides superior conditions for bone regeneration.

## Introduction

Defects of craniofacial bones can lead to significant complications in the appearance and oral function of patients [1]. Scaffold based tissue engineering approaches have shown promise in the reconstruction of bone defects. However, tissue-engineering based bone regeneration continues to face considerable challenges. While ideally porous scaffold materials should mimic the extracellular matrix (ECM) of native tissue in order to ensure adequate nutrient and oxygen diffusion, waste product removal as well as cellular infiltration and vascularization, the scaffold(s) must also have adequate mechanical integrity to cope with its environment, possess an appropriate biodegradation profile and minimise any host inflammatory response during the regenerative process [2–4].

PCL is a highly biocompatible biodegradable polyester with a low degradation rate which is resorbed slowly making it a good candidate for regenerative medicine applications. In this respect, PCL has been shown to have significant potential for bone and cartilage repair [5]. PCL also has sufficient mechanical properties to tolerate stress loads after implantation [6]. The porosity of the scaffold is the most important factor regulating these mechanical properties, the penetration of regenerated tissue and subsequent vascularization. On the other hand, reducing the elastic modulus by increasing the size of pores has also been shown to enhance cell and blood vessels infiltration [7].

According to our previous studies and other reported research, the offset and gradient pore size structured scaffolds provided dense structures that have the advantages of mechanical integrity and high porosity for better cell infiltration in bone tissue engineering [8–11]. The similarity of the gradient scaffold structure to the hierarchical and heterogeneous architecture of native bone, has been shown to be advantageous in overcoming the limitations of scaffolds with homogeneous pore sizes. The gradually increasing pore size from one layer to the next mimics the changes in mineral density from cortical bone to cancellous bone. The most porous region of the gradient structure mimics natural cancellous bone that promotes cell permeability, migration, efficient oxygen /waste diffusion, nutrient supply and vascularization. The denser architecture of the gradient scaffold with the smallest pore size layer acts like native cortical bone which facilitates greater protein adsorption and enhances mechanical support to withstand external loads and maintain and recover their elastic properties after deformation. Sobral et al. showed that gradient structured fused deposition modeling PCL scaffolds had better recovery after mechanical deformation as well as, better osteosarcoma cell distribution compared to homogeneous porous scaffolds [9]. Serra et al. [12] however showed staggered filaments of three-dimensional (3-D) printed polylactic acid and a bioactive CaP glass scaffold decreased the elastic modulus up to 75% in comparison with aligned fibers. Others studies [13, 14] have shown an increase in alkaline phosphatase activity, calcium mineralization and faster new bone formation in compartments with the largest pores of gradient architecture PCL scaffolds. Both groups suggested the reason for improvement was due to a better supply of oxygen and nutrients in the larger pores of the gradient structure.

Melt electrowritten (MEW) scaffolds are solvent-free and possess a very highly ordered and tuneable architecture. Therefore don’t have the limitations of solution electrospinning, namely potential cytotoxicity due to incomplete evaporation of solvents as well as a random fiber orientation resulting in insufficient porosity which may inhibit cell infiltration [15–17]. MEW scaffolds provide predictable filament deposition which creates uniform pore size distribution, orientation and pore interconnectivity [18] resulting in enhanced angiogenesis and cell penetration / ingrowth leading to faster repair of bone defects. This study aims to address the lack of in vivo data assessing the effect of MEW PCL scaffolds with offset and gradient structures on bone regeneration.

## Methods

### Fabrication of scaffolds

**Fig. 1:**
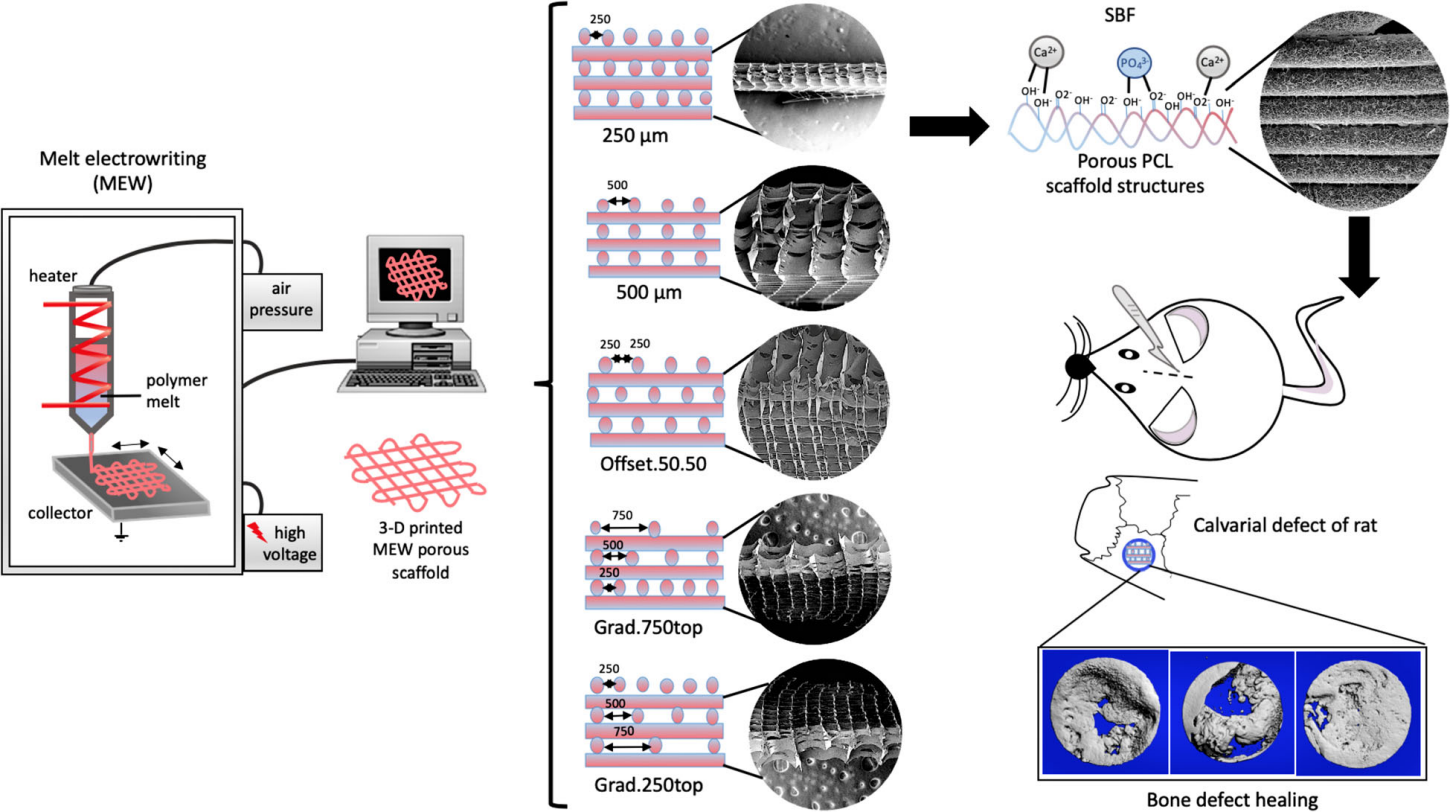
Schematic diagram of experimental procedures and scaffold design used for the bone regeneration study. The left panel shows the custom-built melt electrowriting device. In the middle panel, diagrammatic representation of the different scaffold structures and SEM images of 3D printed scaffolds. The right panel illustrates the uniform hydroxyapatite coating process on the surface of the porous scaffolds by soaking in SBF and subsequent implantation of the scaffolds into a calvarial defect in rats

Based on published in vitro data [10, 19], it is hypothesised that significant bone regeneration can be achieved highly by gradient MEW PCL scaffolds including large pore sizes (250–500-750 μm). To this end, medical grade polycaprolactone was utilized to fabricate membranes according to our published protocol via the technique of MEW [10]. The scaffolds were coated with CaP to improve the hydrophilicity and bioactivity before being implanted into a critical sized osseous defect created in the calvarium of rats (Fig. 1).

### Animals

All animal experimentation was performed as authorized by the Griffith University Animal Ethics Committee (approval # DOH/01/17/AEC). Thirty skeletally mature female Wistar rats (150 ± 20 g) were sourced from the Animal Resource Centre (ARC, Western Australia) and housed in pairs in a temperature controlled animal facility with light/dark cycles (12/12 h). Both food and water was provided ad libitum.

### Surgical procedures

The initial body weight of each animal was recorded before the surgical procedures. General anaesthesia was administered using a Mediquip vaporiser which enabled precise control of the level of anaesthesia. A dosage of Isoflurane (up to 5%) (ProVet, Australia) was used for induction and followed (by 1–3%) to maintain anaesthesia. The rats were kept on a heating pad during surgery and immediately postoperatively in order to maintain body temperature. Any unconscious or sedated animals were held in separate cages without the presence of any other alert and active rats.

**Fig. 2:**
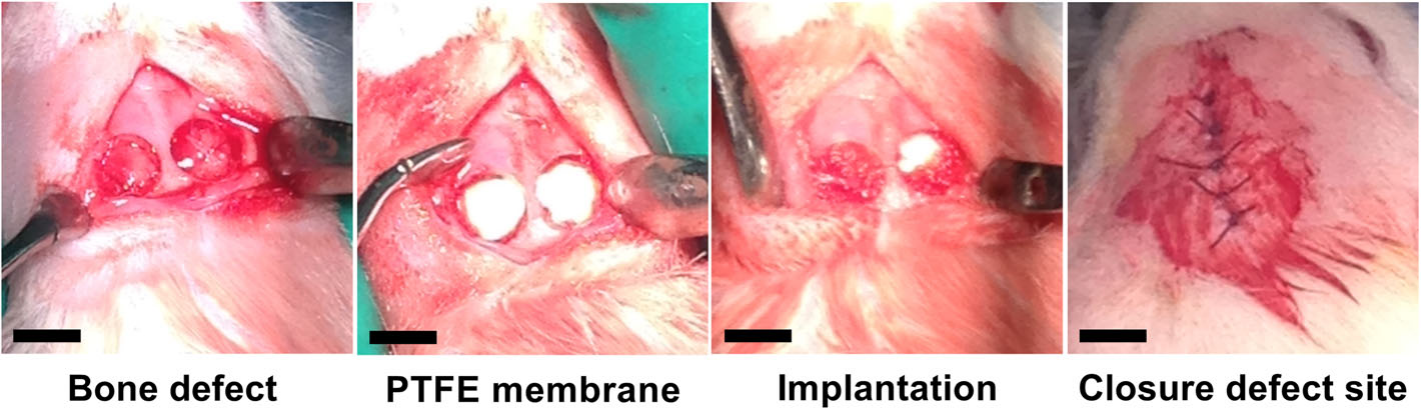
Photographs of the surgical procedures whereby two circular calvarial defects were created in the rat and subsequently filled with PTFE membrane to prevent soft tissue infiltration. Then MEW scaffolds were implanted into the defect site and the defect was closed in layers using sutures. The defects were subsequently left to heal for 4 and 8 weeks (Scale bar = 5 mm)

To assist with post-operative pain management, buprenorphine (0.05 mg/kg) was given by subcutaneous injection at least 15 min prior to surgery. The dorsal part of cranium was then shaved and the skin of the operation site disinfected with Povidone-Iodine (50 mg/mL) (Betadine, Mundipharma BV, Netherland). A sagittal incision was made through the skin over the parietal bone and the periosteum of the calvarium and the cranial vertex was uncovered. After raising the full thickness periosteal flap, 2 circular cranial defects (5 mm) were created in each animal using a trephine bur (external diameter of 5.0 mm) (Komet Dental, Germany) under copious isotonic solution (0.9% saline) irrigation not to damage the underlying blood vessels or the dura mater. An occlusive PTFE membrane (Cytoplast, GBR-200 Barrier Membranes, Osteogenics Biomedical, USA) was then placed on top of the exposed *dura mater* in order to prevent the infiltration of any soft tissue into the defect and away from the newly created osseous defects (Fig. 2).

**Fig. 3:**
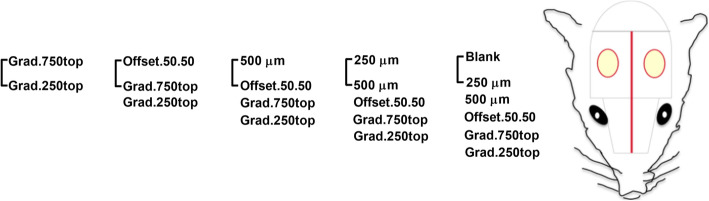
Schematic of the calvarial defects and the combination of MEW scaffolds subsequently placed in vivo. To ensure sufficient material for subsequent analysis, five replicates of each scaffold for both healing time points (4 and 8 weeks) were placed

Each defect received one of the following treatments as shown in Fig. 3: a) Blank (injury without implanted scaffold), b) 250 μm, c) 500 μm, d) Offset.50.50, e) Grad.250top and f) Grad.750top. The soft tissue was then closed using resorbable coated vicryl sutures (Vicryl 5.0, Ethicon, Germany). Animal revival was assisted using pure oxygen, and conscious animals were transferred into a cage until complete recovery. Postoperative analgesia, Buprenorphine (0.01–0.05 mg/kg SC) and prophylactic antibiotic cover, Enrofloxacin (at a dose of 2.5 mg/kg) (Baytril, Bayer UK) were administered subcutaneously. During the postoperative period, the antibiotic delivery continued per os (at a dose of 2.5 mg/kg) per day for 7 days. All drug delivery was performed using a needle (23-gauge) via an intraperitoneal route. Recovery was considered complete when the rats were able to move purposefully in their cage.

The animal’s surgical wound, condition, activity, food intake and any clinical signs of infection during recovery were monitored daily. Following healing periods of 4 and 8 weeks, the animals were euthanized by isoflurane overdose when the scaffolds were retrieved and fixed in paraformaldehyde (PFA) in PBS (4%) for 24 h, at 4 °C for further analysis including Micro-CT, histology and immunohistochemistry.

### μ-CT image acquisition

Micro-CT scans of the recovered calvarial with the scaffold in situ was performed in a μ-CT scanner (μCT40, SCANCO Medical AG, Bruttisellen, Switzerland) at a resolution of 15 μm, a voltage of 55 kV and a current of 150 μA at a greyscale threshold of 150. Three-dimensional images were constructed (frame averaging = 1) and the average density of mineralisation determined using the μ-CT software. New bone volume in the test scaffolds measured in the defect region were determined by subtracting the mean bone volume of the ‘blank’ (without scaffold) negative control.

### Histomorphometric analysis

Samples of the original surgical defect and 2 mm of surrounding tissue were recovered from the surgical site for histologic investigation. Following fixation of the specimens in PFA (4%), they were subsequently decalcified in neutral ethylenediaminetetracetic acid (14%) at PH 10.0 at 4 °C for 4 weeks and then dehydrated in ascending concentrations of ethanol followed by xylene. After that they were embedded in paraffin. The paraffin blocks were trimmed and 6 μm thick sections were prepared and mounted on charged slides to examine the morphology, integration of neighboring tissue into the scaffold, cell type and tissue inflammation by using H&E staining according to manufacturer’s protocol. To quantitate the percentage area of new bone formation for each group, Fiji ImageJ software was used. The average newly formed bone area/total area per group was calculated.

### IHC staining

Serial paraffin sections were de-waxed in xylene and rehydrated in an ethanol gradient and then water. Antigen retrieval was performed by applying a heat-induced epitope incubation in citrate buffer (10 mM) (PH = 6) at 95 °C for 5 min. Endogenous peroxidase activity was quenched with hydrogen peroxide (3%) for 5 min at RT to reduce endogenous peroxidase activity, and any other potential non-specific binding sites were blocked with Background SNIPER (Biocare Medical, cat.BS966L) for 10 min at RT. The sections were subsequently incubated with the following primary antibodies at 4 °C overnight: anti-Col I antibody (Abcam, ab34710, diluted 1:400), anti-ALP antibody (Abcam, ab108337, diluted 1:250), anti-BMP-2 antibody (Abcam, ab14933, diluted 1:400), anti-Ocn antibody (Abcam, ab13420, diluted 1:250), anti-Opn antibody (Abcam, ab8448, diluted 1:200), anti-CD34 antibody (Abcam, ab81289, diluted 1:200), anti-CD105 antibody (Abcam, ab231673, diluted 10 μg/ml), anti-VEGF antibody (Abcam, ab2349, diluted 1/100), anti-vWF (Abcam, ab6994, diluted 1/800). After rinsing with PBS, the sections were labelled with the corresponding HRP secondary antibodies at RT. The sections were visualised with 3,3-diaminobenzidine (DAB solution; DAKO liquid DAB, ref.: K3468). Mayer’s hematoxylin (Thermo Scientific) was used for nuclei staining at RT. The slides were dehydrated, and the coverslips were mounted onto the slides. All the slides for each antibody were stained at the same time. The soft tissue that did not express the target antigen was considered as negative control and the calvarial bone sample as positive control. The sections were visualised by an Aperio digital pathology slide scanner (Leica Biosystems Inc., USA).

A semi-quantitative analysis of the immunostaining results was performed using Fiji ImageJ software. The staining intensity was assigned a score from 0 to 6 (with 0 indicating a lack of brown immunoreactivity, 1 = 0–4%, 2 = 5–20%, 3 = 21–40%, 4 = 41–60%, 5 = 61–80% and 6 = 81–100%) and the overall score after adjusting for background (isotype staining) was used for subsequent statistical analysis. The mean from three slide sections in each treatment group was used.

### Statistical analysis

All data were expressed as mean ± standard deviation. Comparison between groups were analysed by analysis of variance (ANOVA, post hoc test: Tukey). The statistical software Prism 8.0 for windows was used for calculations and a *p* value < 0.05 was considered statistically significant.

## Results

### Micro-CT (μ-CT) evaluation of newly formed bone

**Fig. 4:**
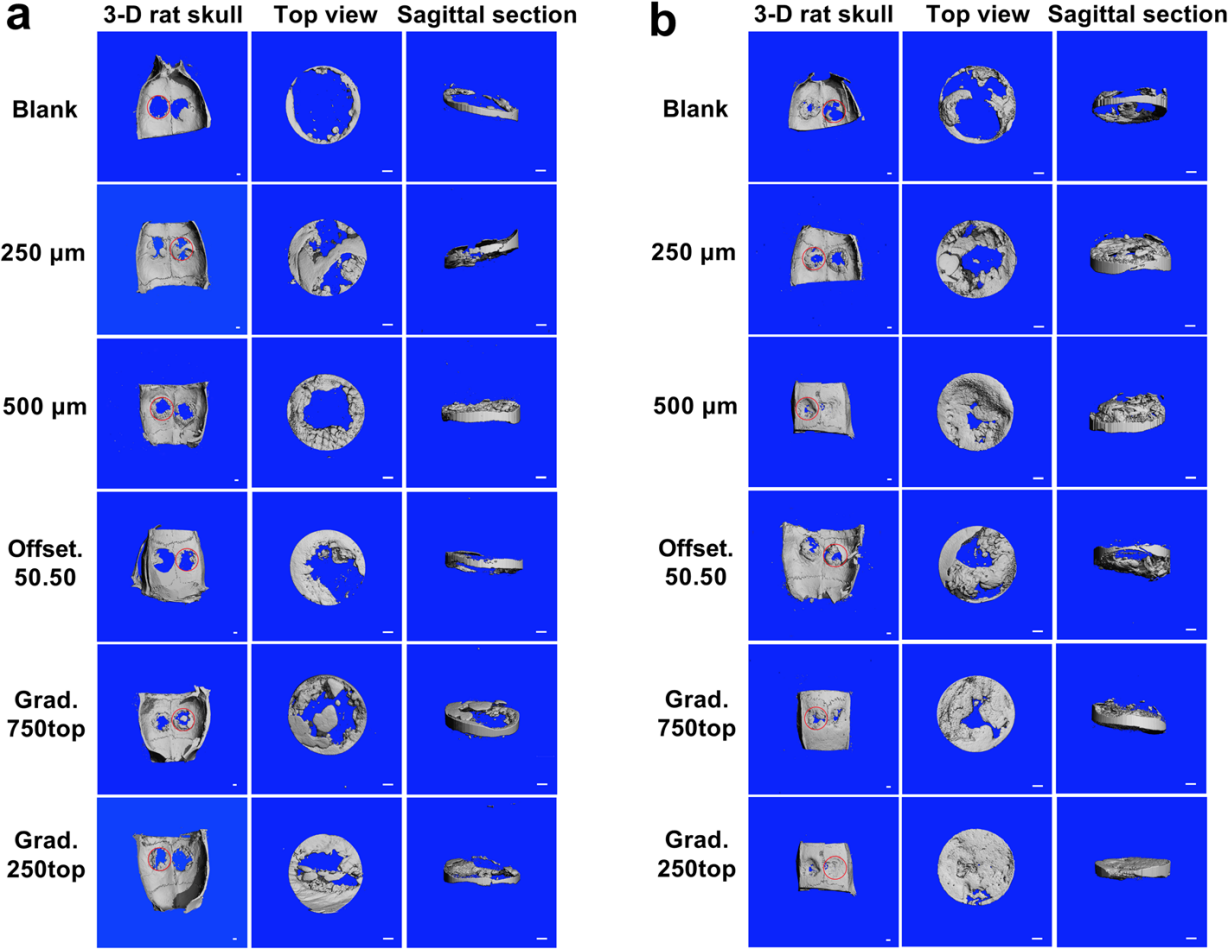
3-D reconstructed Micro-CT image analysis showing the degree of bone repair in the different MEW PCL scaffolds implanted into the rat calvarial, a) 4 weeks post implantation and b) 8 weeks post-surgery. Top and sagittal defect views are of the area indicated by the dashed red line (Scale bar = 1 mm)

Figure 4 shows the topographic features of the newly formed bone in the scaffold-implanted calvarial defects. Images were acquired via reconstruction of the μ-CT scans using the scanner software. In the control defect, the 5 mm diameter defect was shown to be a critical-sized defect as a very limited amount of new bone was regenerated after 4 and 8 weeks of healing. Any new bone was found to circle the primary bone defect. Defects filled with scaffolds with a pore size of 500 μm and the offset.50.50 structures revealed that the new bone was mainly distributed on the periphery of the implanted scaffolds rather than the central region of the porous constructs. Whilst, in 250 μm and both gradient (250top, 750top) scaffolds, the new bone formation was not limited surrounding the defect area and the regeneration increased towards the cavity centre after 4 weeks (Fig. 4a). The bone images indicated that the residual material scaffold was diminished by gradual new bone growth in scaffold groups following 4–8 weeks post-implantation. At 8 weeks post-implantation, the grad.250top indicated a high degree of new bone formation compared to the other scaffold groups (Fig. 4b). In contrast, other groups showed incomplete bone repair following 8 weeks implantation with more empty space.

**Fig. 5:**
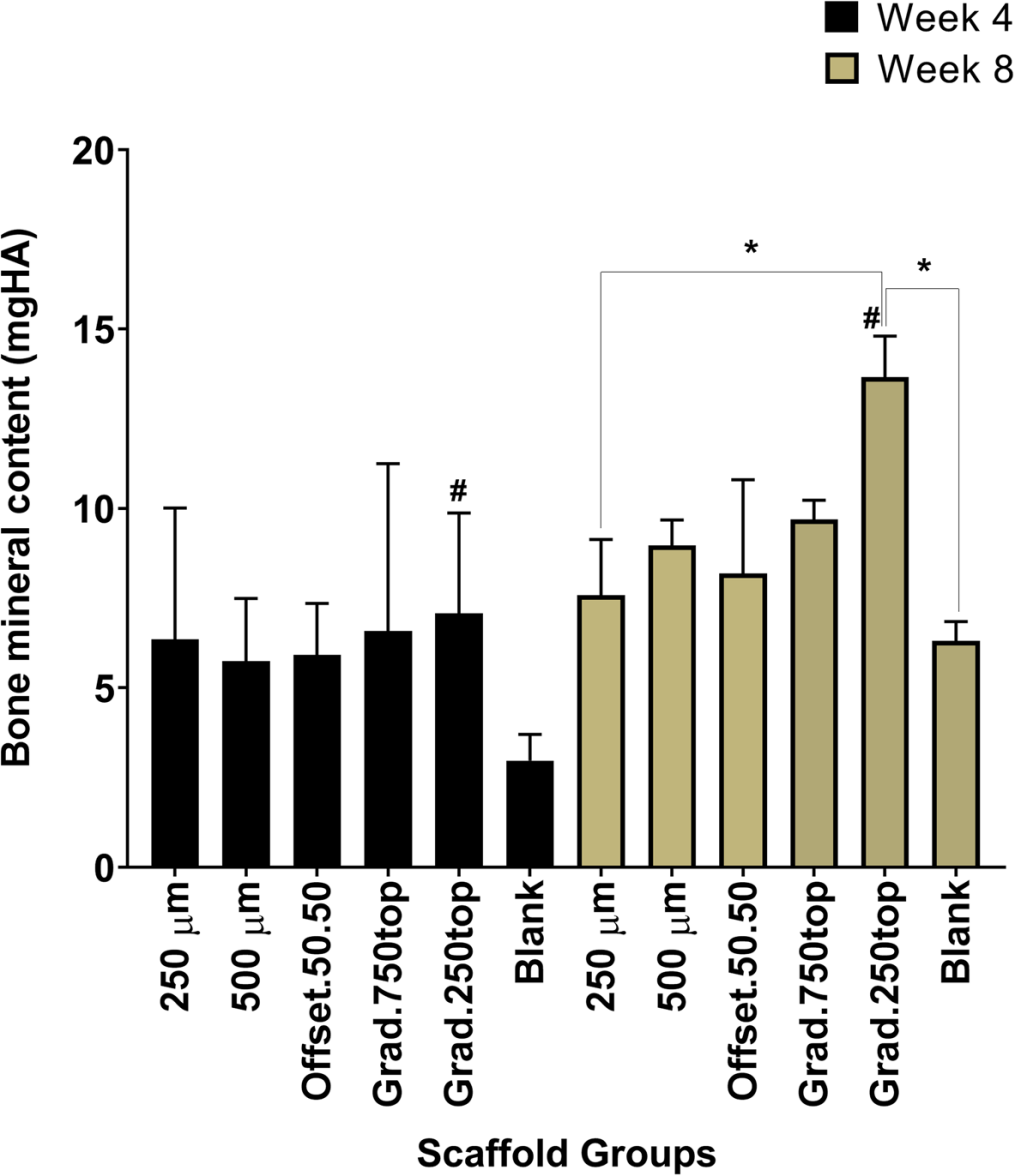
Quantitative micro-CT data analysis of BV/TV ratio. A significant difference in the BV/TV ratio was seen between grad.250top and the 250 μm scaffolds and the control after 8 weeks post-implantation, (number of samples/ group = 5) (* *p* ≤ 0.025). (#) indicates a significant difference between grad250top scaffold groups at the different time points of 4 and 8 weeks (*p* ≤ 0.034)

The new bone volume was also measured by quantitative analysis (Fig. 5). The BV/TV ratio showed a time-dependent increase in all the groups; however, this was only significant for grad.250top scaffolds at the two different time points compared to other groups (*p* ≤ 0.034). The highest BV/TV ratio was found in the grad.250top scaffold following 8 weeks post-implantation which was significant compared to 250 μm scaffold and the blank (*p* ≤ 0.025) (Fig. 5).

### Histological assessment

**Fig. 6:**
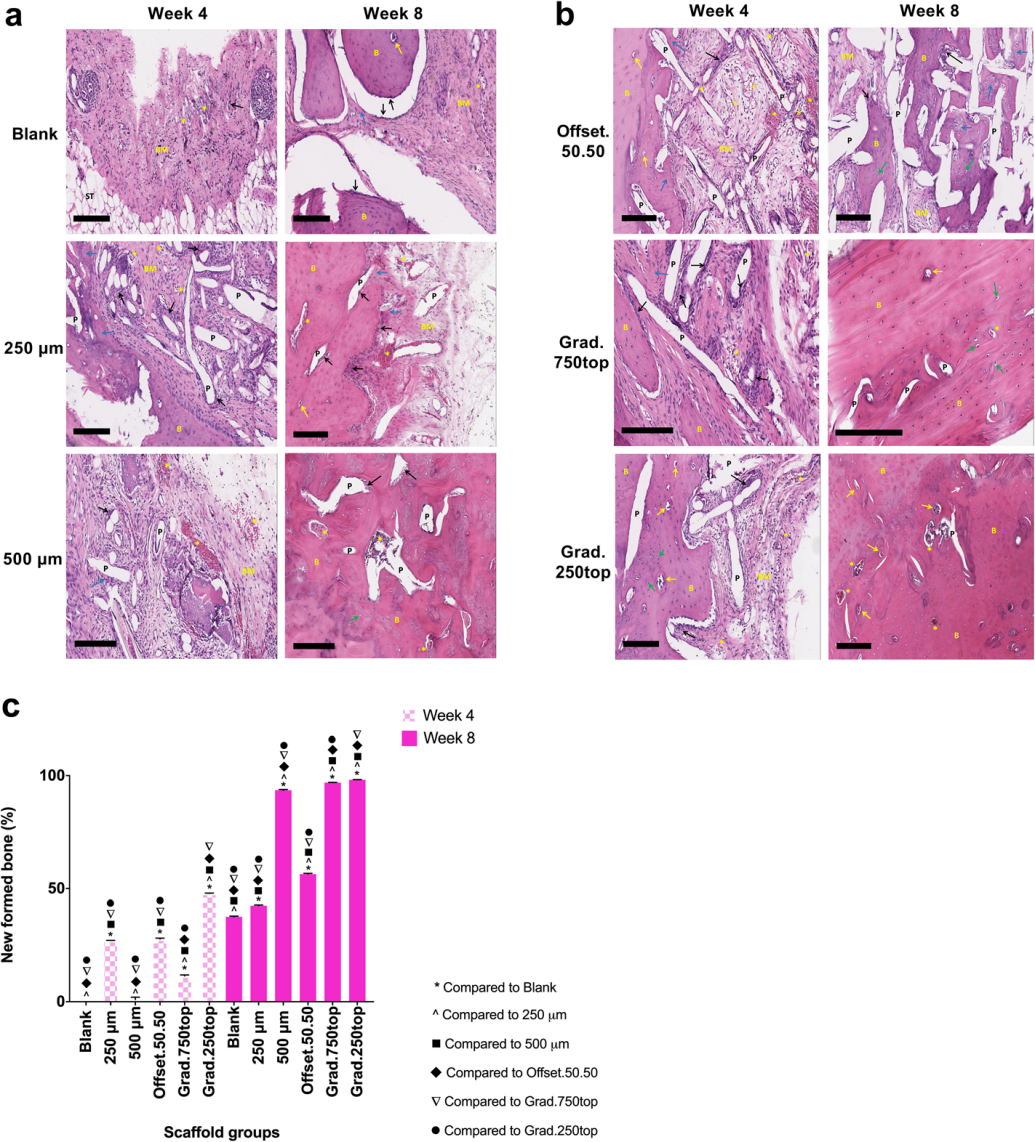
Microscope images of H&E stained tissue sections of MEW PCL scaffolds implanted in rat calvarial defects, a) 4 and b) 8 weeks (top and bottom panels respectively) after surgery. B: new bone; Black arrow: osteoblasts; BM: newly formed bone matrix; ST: Soft tissue; P: pore remaining implanted scaffold material; Blue arrow: new bone forming; Green arrow: osteocytes; Yellow arrow: osteocytes in lacuna; Asterisk: blood vessel; The black scale bar represents 200 μm. c) The percentage of newly formed bone for each group was calculated 4 and 8 weeks postoperatively using Fiji ImageJ software. The values expressed are the mean ± SD (*n* = 3). Statistically significant differences between the mean %bone for the treatment groups when compared to each other were determined by ANOVA (Significant: *, ^, ■, ♦, ▼, ● all *p* < 0.0001)

Figure 6 shows representative haematoxylin and eosin (H&E) staining images of the retrieved samples after 4 and 8 weeks of healing. In the control group (without scaffold) only either bone matrix or soft tissue were observed with a few osteoblast cells (Fig. 6a). Little mature bone tissue was observed within the blank and 250 μm scaffold groups, rather immature osteoid like tissue containing prominent newly formed bone matrix tissue containing osteoblasts (Fig. 6a). At 4 weeks, new bone tissue observed in the offset.50.50 and gradient grafts was denser at the edges towards the centre of the defect area. In grad.250top constructs, the new bone was formed around the PCL scaffolds and ‘osteocytes in lacuna’ like structures were observed. On the other hand, towards the centre of the scaffold more newly-formed bone matrix accompanied with osteoblasts that were located close to the pores of the scaffold (Fig. 6b). Visual evaluation of the amount of newly formed bone in this group were confirmed by the quantitative H&E staining analysis (Fig. 6c).

After 8 weeks healing, H&E staining demonstrated newly formed bone tissue was produced and distributed throughout the scaffolds, especially the gradient and 500 μm scaffolds (Fig. 6a, b, c). The grad.250top scaffold showed high bone content and the pores of the scaffold were completely filled with compact bone (Fig. 6b, c). The pores observed in the images were created by the xylene dissolving the PCL scaffold fibres during processing as the pores were infiltrated with the new bone. The bone matrix was reduced in the 250 μm, offset.50.50 scaffolds and the blank in contrast to that at 4 weeks (Fig. 6a and b). While the 500 μm scaffold showed bone regeneration after 8 weeks, there were still empty pores that needed to be fully covered (Fig. 6a, c) compared to the gradient scaffolds, suggesting that gradient scaffolds produced more new bone tissue compared to the other groups (Fig. 6b, c).

### Immunihistochemistry (IHC) staining

#### Collagen I (Col I) Immunostaining

**Fig. 7:**
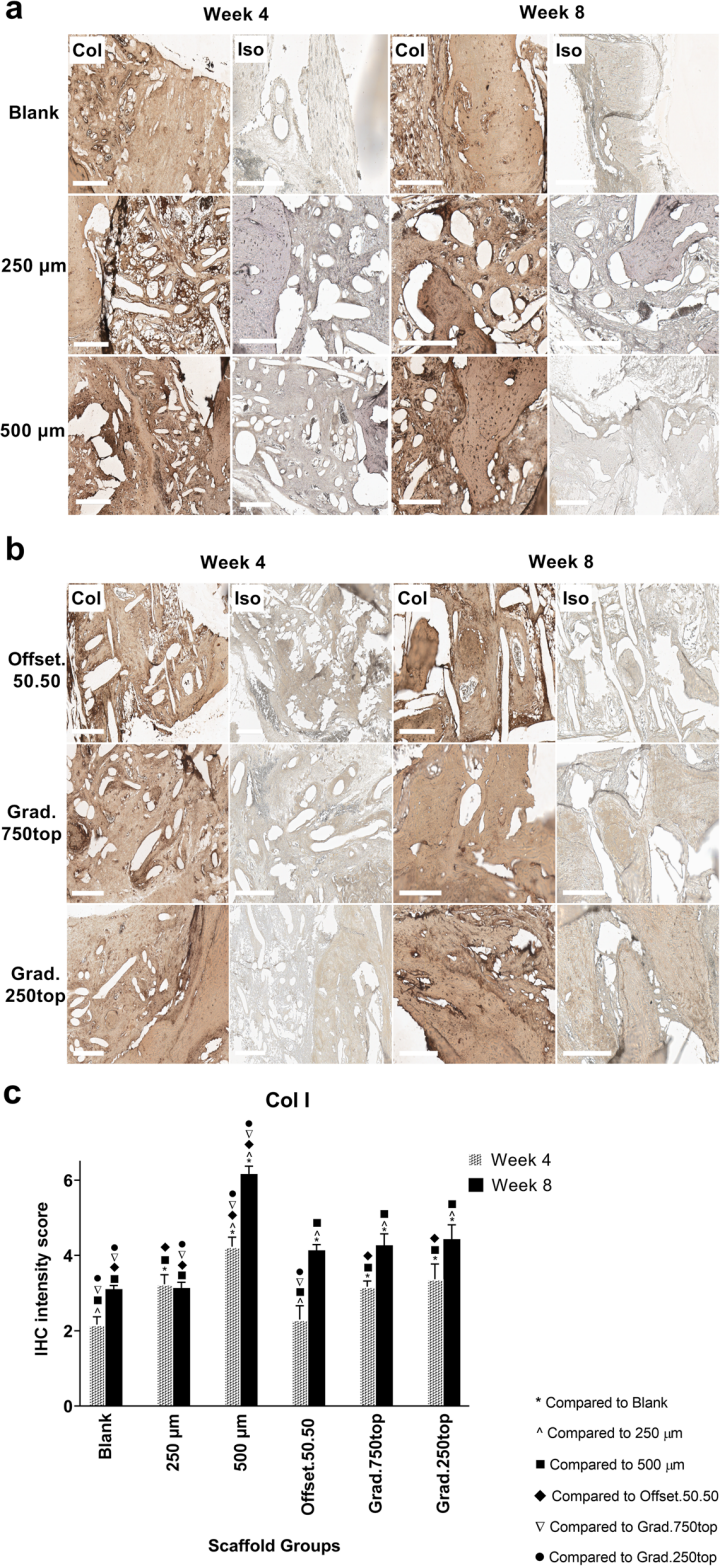
IHC analysis of Col I in calvarial defects from rats 4 and 8 weeks post-implantation of MEW PCL scaffolds. a) Blank (defect control without implanted scaffold), 250 μm, 500 μm. b) Offset.50.50, Grad.750top, Grad.250top. Col: collagen type I staining, Iso: Isotype control. The white scale bar represents 200 μm. c) Quantitative analysis of Col I staining at weeks 4 and 8. The values expressed are the mean ± SD (*n* = 3). Statistically significant differences between mean Col I intensity scores for the treatment groups when compared to each other were determined by ANOVA (Significant: * *p* ≤ 0.0001, ^ *p* = 0.004, ■ *p* ≤ 0.0001, ♦ *p* = 0.002, ▼*p* = 0.002, ● *p* ≤ 0.0004)

Immunohistochemical analysis displayed markedly strong staining of Col I throughout the constructs in all groups 8 weeks after implantation. No positively stained tissues were seen in the isotype control group (Fig. 7a, b), however significantly (*p* ≤ 0.0001) more Col I production was observed in the 500 μm scaffold after 4 weeks compared to the other groups (Fig. 7c). Intense staining in the 500 μm, offset.50.50 and gradient scaffolds 8 weeks post-surgery suggested the expression of Col I increased further with healing time in these groups (*p* ≤ 0.0001, *p* = 0.002, *p* = 0.0004 respectively) (Fig. 7c).

#### Alkaline phosphatase (Alp) Immunostaining

**Fig. 8:**
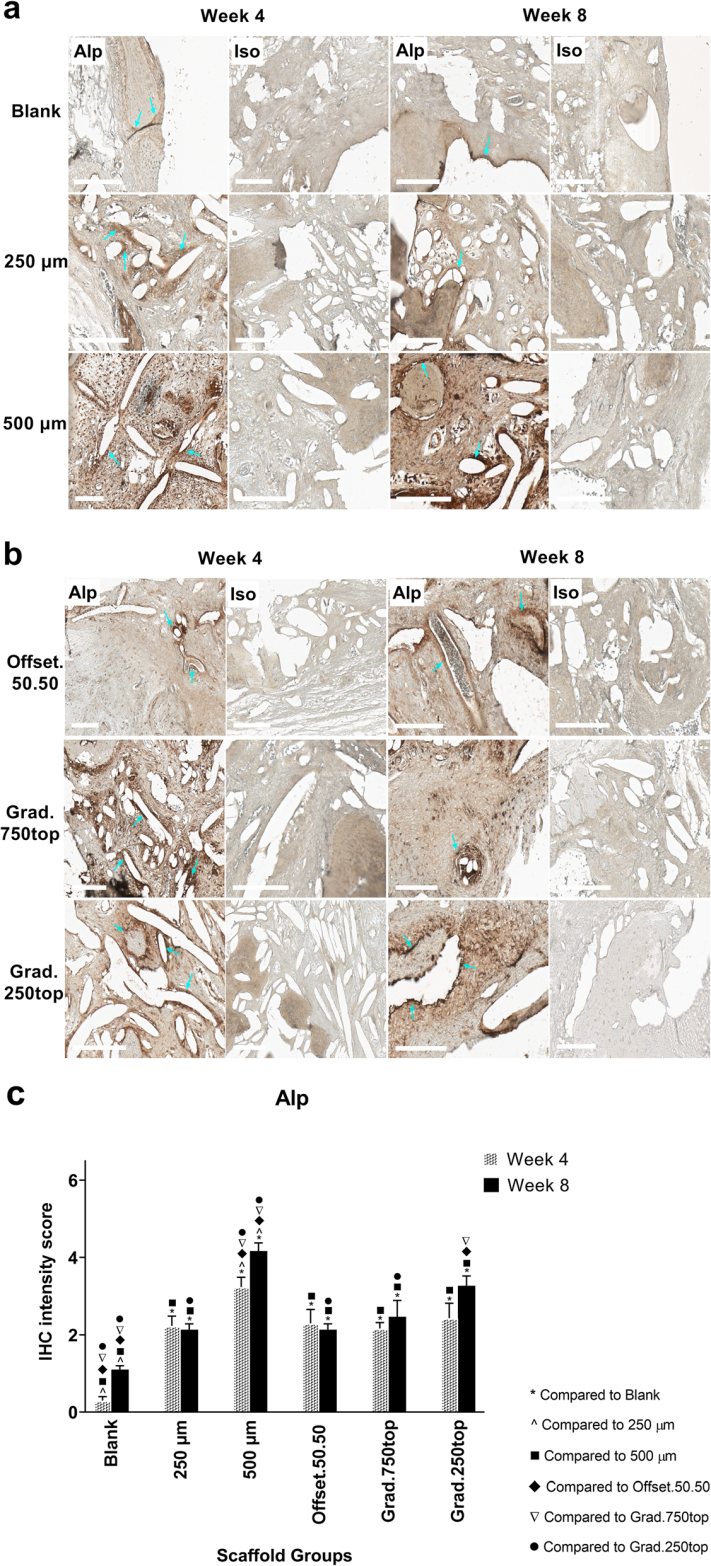
IHC analysis of Alp in calvarial defects from rats 4 and 8 weeks post-implantation of MEW PCL scaffolds. a) Blank (defect control without implanted scaffold), 250 μm, 500 μm. b) Offset.50.50, Grad.750top, Grad.250top. Alp: alkaline phosphatase staining, Iso: Isotype control. The white bar represents 200 μm. c) Quantitative analysis at weeks 4 and 8. The values expressed are the mean ± SD (*n* = 3). Statistically significant differences between mean Alp intensity scores for the treatment groups when compared to each other were determined by ANOVA (Significant: * *p* < 0.0001, ^ *p* = 0.002, ■ *p* < 0.0001, ♦ *p* = 0.002, ▼*p* = 0.001, ● *p* = 0.009)

IHC analysis of the stained sections showed the overall trend in Alp expression (apart from 250 μm and offset.50.50) was to increase between weeks 4 and 8. This was most pronounced in the 500 μm scaffold group (Fig. 8c). The positive staining areas were mostly localized at the pore margins or at the borderline of newly formed bone tissue as shown (blue arrow) in all the scaffold groups (Fig. 8a, b).

#### Bone morphogenetic protein-2 (Bmp-2) Immunostaining

**Fig. 9:**
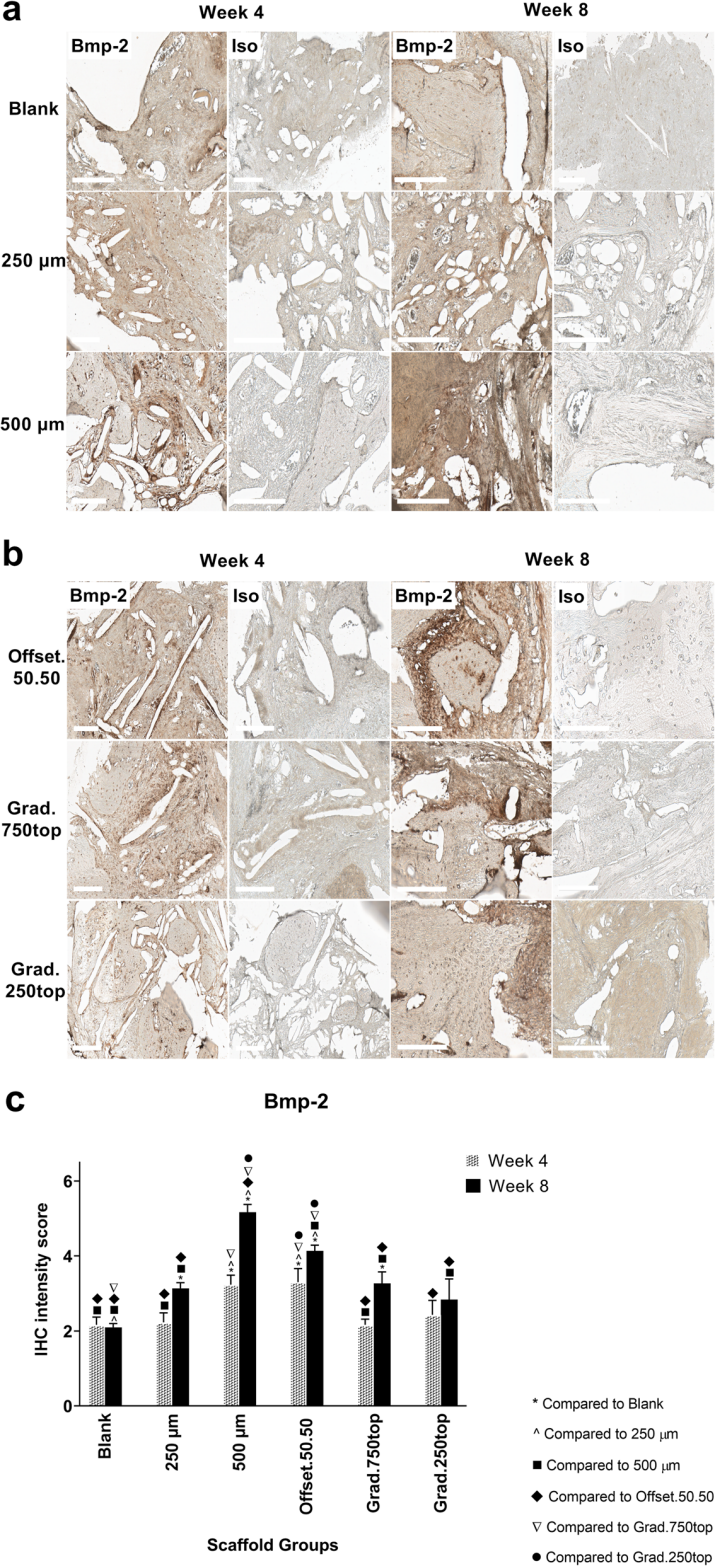
IHC analysis of Bmp-2 in calvarial defects from rats 4 and 8 weeks post-implantation of MEW PCL scaffolds. a) Blank (defect control without implanted scaffold), 250 μm, 500 μm. b) Offset.50.50, Grad.750top, Grad.250top. Bmp-2: bone morphogenic protein-2 staining, Iso: Isotype control. The white bar represents 200 μm. c) Quantitative analysis at weeks 4 and 8. The values expressed are the mean ± SD (*n* = 3). Statistically significant differences between Bmp-2 intensity scores for the treatment groups when compared to each other were determined by ANOVA (Significant: * *p* < 0.0001, ^ *p* = 0.009, ■ *p* = 0.005, ♦ *p* = 0.002, ▼*p* = 0.005, ● *p* < 0.0001)

IHC analysis showed the 500 μm scaffold at 8 weeks of treatment displayed the highest Bmp-2 expression compared to the other groups (Fig. 9a, c). However, there was also strong staining observed between weeks 4 to 8 for the offset.50.50 sample (Fig. 9c). Quantitative IHC analysis showed a significant difference between the two healing time points of 4 and 8 weeks for the 250 μm, 500 μm and grad.750top scaffolds (*p* = 0.004, *p* < 0.0001 and *p* = 0.005 respectively) (Fig. 9c).

#### Osteocalcin (Ocn) Immunostaining

**Fig. 10:**
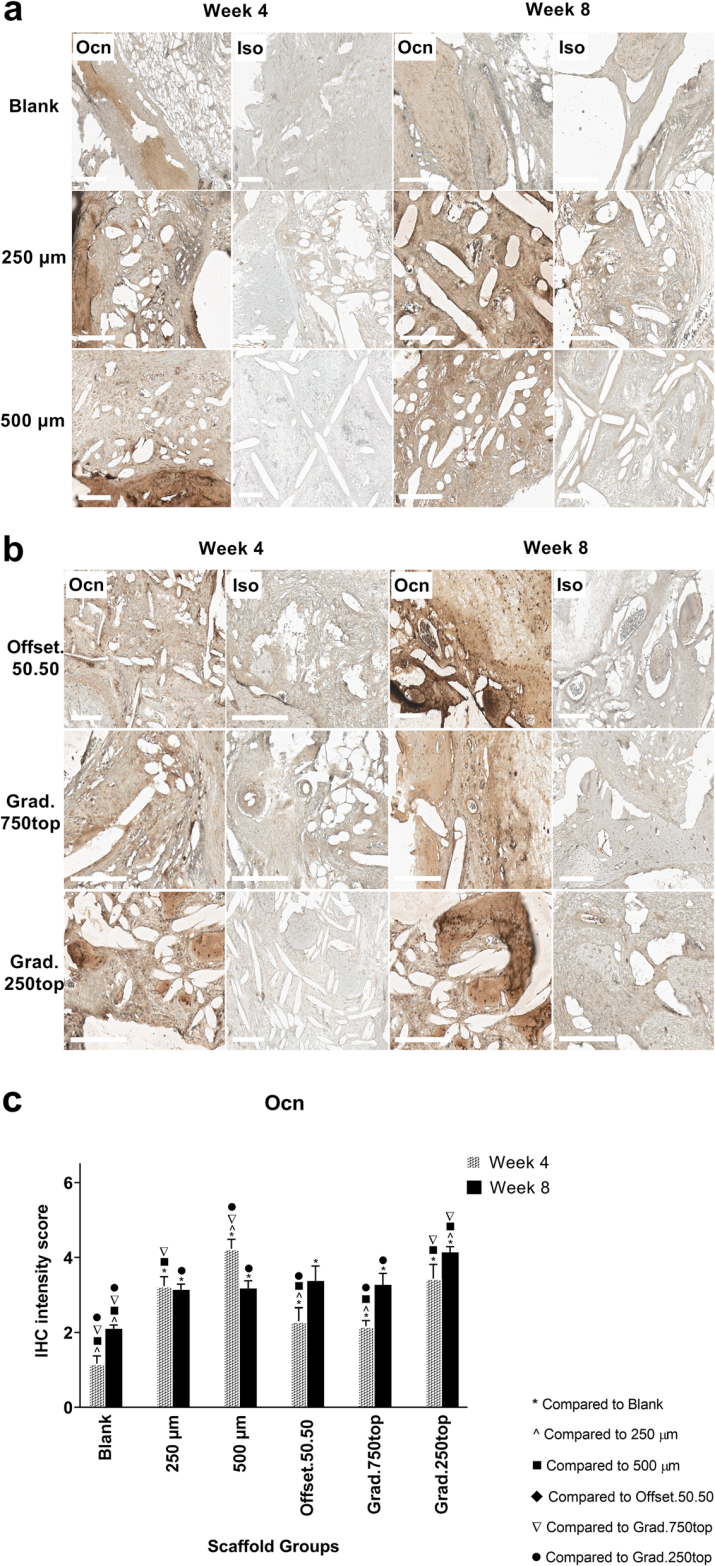
IHC analysis of Ocn in calvarial defect of rat 4 and 8 weeks of post-implantation of the MEW PCL scaffolds. a) Blank (defect control without implanted scaffold), 250 μm, 500 μm. b) Offset.50.50, Grad.750top, Grad.250top. Ocn: osteocalcin staining, Iso: Isotype control.. The white bar represents 200 μm. c) Quantitative analysis at weeks 4 and 8. The values expressed are the mean ± SD (*n* = 3). Statistically significant differences between mean Ocn intensity scores for the treatment groups when compared to the other groups were determined byby ANOVA (Significant: * *p* < 0.0001, ^ *p* = 0.004, ■ *p* < 0.0001, ♦ *p* = 0.006, ▼*p* < 0.0001, ● *p* = 0.002)

The IHC results showed specific expression of Ocn in the treatment groups (Fig. 10a, b). The intensity of Ocn staining was increased in the grad.250top group compared with the other scaffold groups following 4 weeks of healing (Fig. 10c). Similar expression levels of Ocn in the 500 μm scaffold were attributed to the original old bone and not the newly formed bone at 4 weeks (Fig. 10a). Quantitative analysis of the immunohistochemistry results for Ocn showed the staining intensity was significantly greater in the grad.250top scaffold compared to the others after 8 weeks indicating greater osteogenic activity in the gradient structure (Fig. 10c).

#### Osteopontin (Opn) Immunostaining

**Fig. 11:**
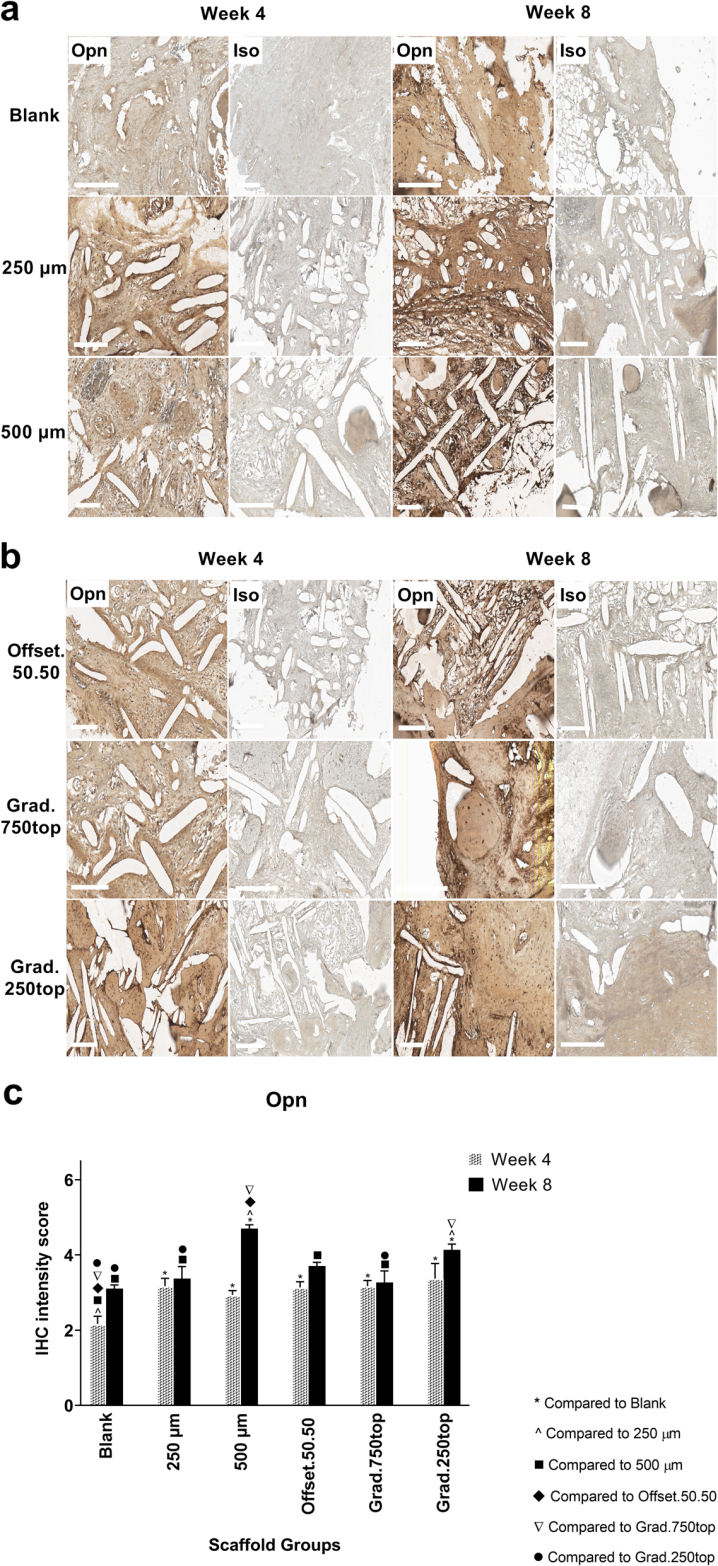
IHC analysis of Opn in calvarial defect of rat 4 and 8 weeks of post-implantation of the MEW PCL scaffolds. a) Blank (defect control without implanted scaffold), 250 μm, 500 μm. b) Offset.50.50, Grad.750top, Grad.250top. Opn: Osteopontin staining, Iso: Isotype control. The white bar represents 200 μm.c) Quantitative analysis at weeks 4 and 8. The values expressed are the mean ± SD (*n* = 3). Statistically significant differences between mean Opn intensity scores for the treatment groups when compared to the other groups were determined by ANOVA (Significant: * *p* < 0.0001, ^ *p* = 0.0004, ■ *p* < 0.0001, ♦ *p* = 0.0006, ▼*p* = 0.0004, ● *p* = 0.002)

IHC analysis showed that Opn was more strongly detected for all treatment groups after 8 weeks of implantation compared with that at week 4 (Fig. 11a, b). Higher expression was localized in more mineralized areas of the new bone tissue in the 500 μm, gradient and offset scaffold structures in comparison to the other tissues (Fig. 11a, b, c). However, more intense staining was also was found at the margins of the pores of these scaffolds.

#### CD34 Immunostaining

**Fig. 12:**
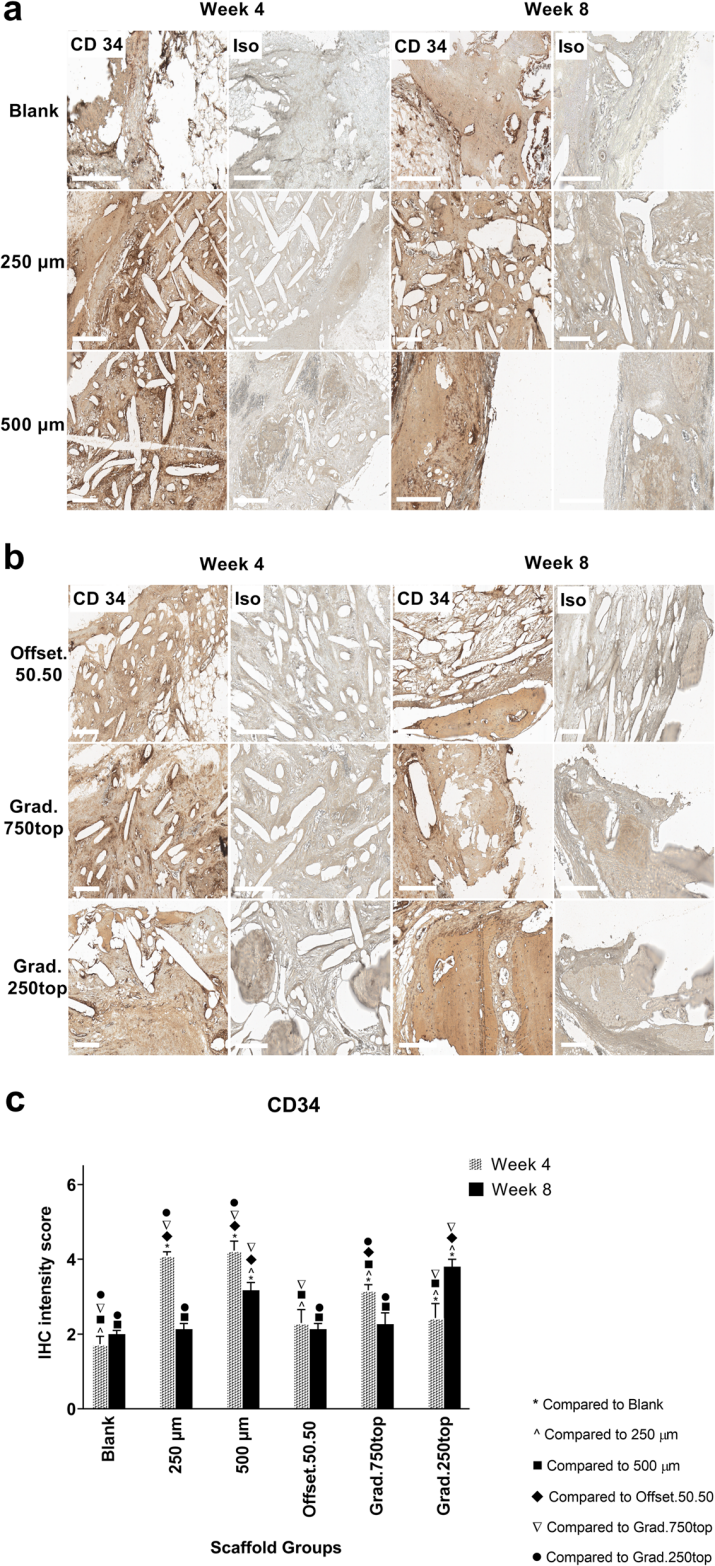
IHC analysis of CD34 in calvarial defect of rat 4 and 8 weeks of post-implantation of the MEW PCL scaffolds. a) Blank (defect control without implanted scaffold), 250 μm, 500 μm. b) Offset.50.50, Grad.750top, Grad.250top. CD34: CD34 staining, Iso: Isotype control. The white bar represents 200 μm. c) Quantitative analysis at weeks 4 and 8. The values expressed are the mean ± SD (*n* = 3). Statistically significant differences between mean CD34 intensity scores for the treatment groups when compared to the other groups were determined by ANOVA (Significant: * *p* < 0.0001, ^ *p* < 0.0001, ■ *p* = 0.0007, ♦ *p* < 0.0001, ▼ *p* < 0.0001, ● *p* < 0.0001)

CD34 staining was also detected in all the treatment groups compared to isotype control (Fig. 12a, b). The intensity of staining was almost the same in all the groups except 500 μm and grad.250top after 8 weeks of surgery (Fig. 12c). CD34 positive cells were more localized either around the pores in the 250, 500 μm and both gradient scaffolds (Fig. 12a), or close to the primary host bone tissue and bone-soft tissue interface in the 250 μm scaffolds after 4 weeks, or in soft tissue surrounded by the new bone or pores of the 500 μm, offset.50.50 and gradient scaffolds 8 weeks after implantation (Fig. 12a, b).

#### Endoglin (CD105) Immunostaining

**Fig. 13:**
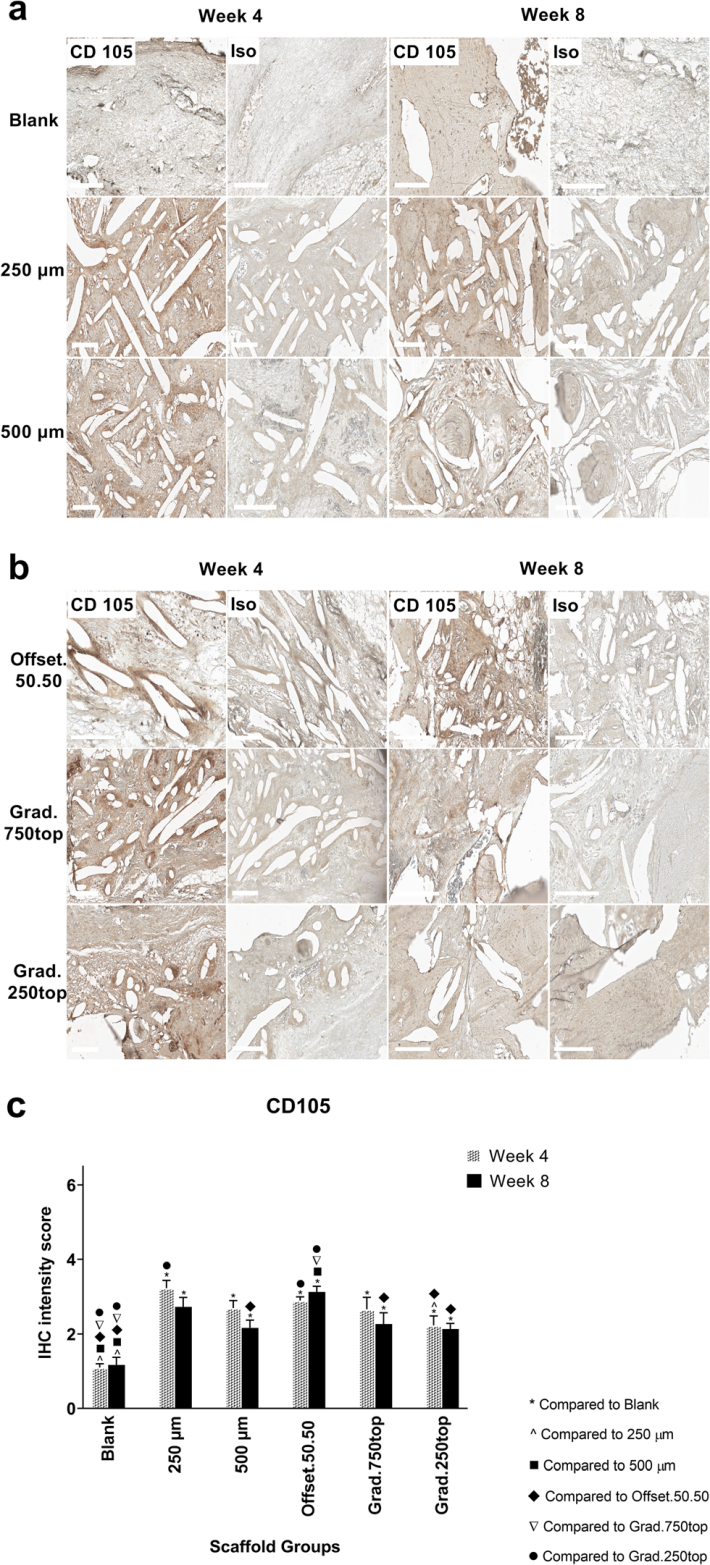
IHC analysis of CD105 in calvarial defect of rat 4 and 8 weeks of post-implantation of the MEW PCL scaffolds. a) Blank (defect control without implanted scaffold), 250 μm, 500 μm. b) Offset.50.50, Grad.750top, Grad.250top. CD105: endoglin staining, Iso: Isotype control. The white bar represents 200 μm. c) Quantitative analysis at weeks 4 and 8. The values expressed are the mean ± SD (*n* = 3). Statistically significant differences between mean CD105 intensity scores for the treatment groups when compared to each other were determined by ANOVA (Significant: * *p* < 0.0001, ^ *p* < 0.0001, ■ *p* = 0.0006, ♦ *p* = 0.002, ▼*p* = 0.002, ● *p* = 0.0006)

A similar tendency of CD105 expression as for CD34 expression was observed regarding localization of positive cells surrounding the scaffold pores. CD105 positive cells however were accumulated more around the pores in all the scaffold groups after 4 weeks instead of bone matrix tissue as seen for CD34 (Fig. 13a, b). In addition, strong staining was observed close to the pores in the scaffolds after 4 weeks of treatment (Fig. 13a, b). In comparison to CD34, more intense staining of CD105 was detected in the offset50.50 and 250 μm scaffolds (Fig. 13c).

#### Vascular endothelial growth factor (VEGF) Immunostaining

**Fig. 14:**
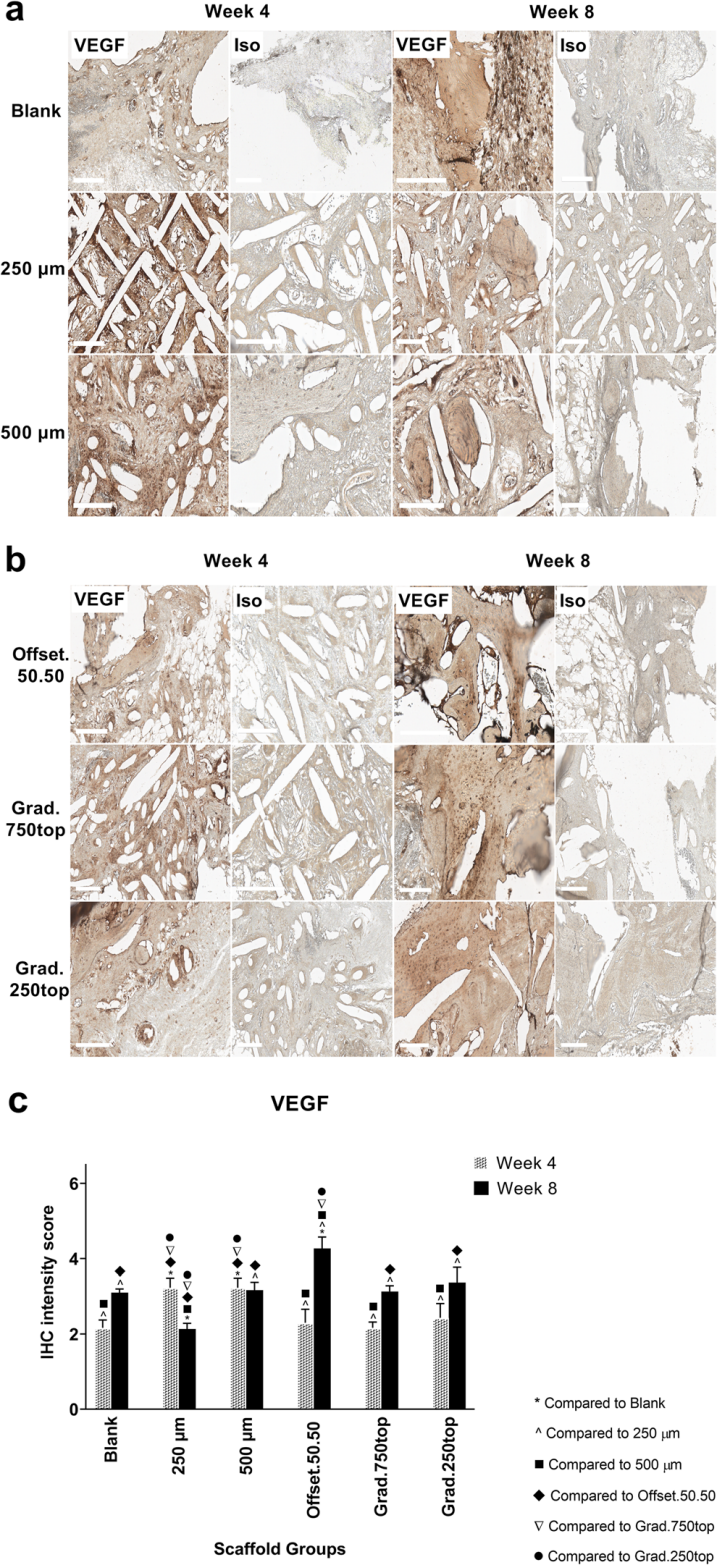
IHC analysis of VEGF, in calvarial defect of rat 4 and 8 weeks of post-implantation of the MEW PCL scaffolds. a) Blank (defect control without implanted scaffold), 250 μm, 500 μm. b) Offset.50.50, Grad.750top, Grad.250top. VEGF: vascular endothelial growth factor staining, Iso: Isotype control. The white bar represents 200 μm. c) Quantitative analysis at weeks 4 and 8. The values expressed are the mean ± SD (*n* = 3). Statistically significant differences between mean VEGF intensity scores for the treatment groups when compared to the other groups were determined by ANOVA (Significant: * *p* = 0.002, ^ *p* = 0.009, ■ *p* = 0.002, ♦ *p* = 0.001, ▼*p* = 0.042, ● *p* = 0.037)

The expression of VEGF in the recovered PCL implant samples showed that VEGF expression was most intense in the treatment groups (Fig. 14a, b). Intense staining was observed in 250 μm and 500 μm after 4 weeks compared to other scaffold groups (Fig. 14c). However, 8 weeks following implantation, strong staining was seen in the offset.50.50 which was localized around the pores and the bone matrix tissue (Fig. 14b, c), followed by the blank, 500 μm and gradient scaffolds which indicated newly formed bone tissue (Fig. 14c).

#### von Willebrand factor (vWF) Immunostaining

**Fig. 15:**
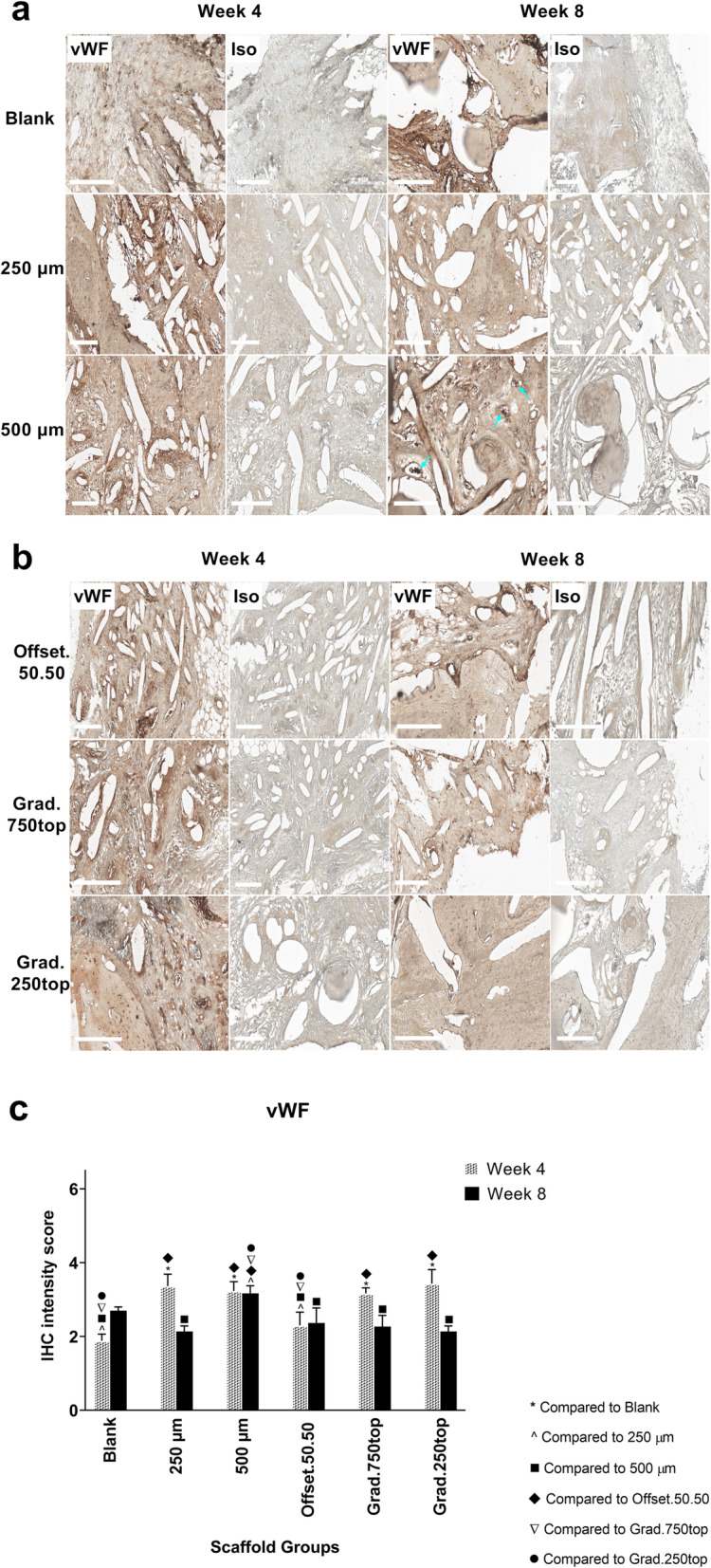
IHC analysis of vWF in calvarial defect of rat 4 and 8 weeks of post-implantation of the MEW PCL scaffolds. a) Blank (defect control without implanted scaffold), 250 μm, 500 μm. b) Offset.50.50, Grad.750top, Grad.250top. vWF: von Willebrand Factor staining, Iso: Isotype control. The white bar represents 200 μm. c) Quantitative analysis at weeks 4 and 8. The values expressed are the mean ± SD (n = 3). Statistically significant differences between mean vWF intensity scores for the treatment groups when compared to the other groups were determined by ANOVA (Significant: * *p* < 0.0001, ^ *p* = 0.002, ■ *p* = 0.003, ♦ *p* = 0.001, ▼*p* = 0.015, ● *p* = 0.003)

The IHC analysis of vWF expression demonstrated that all the positive groups had less immunoreactivity for vWF compared to VEGF protein (Fig. 15c). Analysis of IHC images confirmed more vWF staining in all treatment groups at 4 weeks rather than 8 weeks post-implantation (Fig. 15c). vWF IHC showed new distinct vessels in the 500 μm scaffold 8 weeks following treatment (blue arrow) (Fig. 15a) however this was not significant compared to other groups (Fig. 15c).

## Discussion

The overall aim of this study was to investigate in vivo, an appropriate design structure of porous MEW scaffolds that could stimulate osteogenesis. The response of bone to implanted scaffolds and its integration are influenced by the scaffold surface composition [20]. Indeed studies have shown some bone regeneration could be obtained in the absence of seeded cells [21]. In this study, CaP coated scaffolds in the form of hydroxyapatite were implanted in vivo to regenerate bone defects. This followed from previous in vitro studies which demonstrated that the incorporation of hydroxyapatite particles into the scaffolds improved their mechanical properties and had a beneficial effect on cell-mediated mineralization and bone formation [22, 23]. In addition, materials without bioactive surfaces have been shown to lead to longer recovery time for bone repair due to a lack of recognition sites for bone*-*bonding ability in non-coated surface scaffolds [24]. Li et al. also demonstrated scaffolds with more roughness on the surface enhanced the surface area of the scaffolds which facilitated bone formation [25]. A balance between architecture and mechanical properties of the scaffolds is essential for effective bone regeneration. Scaffolds need to maintain sufficient porosity to allow vascularization, cell penetration and surrounding tissue ingrowth, as well as have adequate mechanical strength similar to that of natural bone, to prevent premature failure of the implants [26, 27]. One of the most promising methods to control scaffold pore size and the level of porosity is MEW [10]. Although open-cell pores might be more applicable to promote osteogenesis due to better access of oxygen and new bone development into the porosities as well as better vasularization through the channels created by pores connections [28], the type of pores obtaining by MEW technique are the closed-cells pores without interconnectivity with each other and enclosed by the pore walls [29].

A 5 mm calvarial defect was used in our study to evaluate new bone formation. Although some studies have suggested a > 6 mm defect to be ‘critical-sized’ i.e. defects smaller than this size can regenerate spontaneously, previous studies by our group have shown 5 mm to be critical [30]. Moreover a number of studies have chosen a 5 mm defect as a standard bilateral skull defect in the rat to minimize the animal numbers used and meet the 3R criteria (Reduction, Replacement, Refinement) [31, 32]. Also, a defect of the cranium is a commonly used model to evaluate bone reconstruction due to the absent blood supply, lack of muscle tissue and poor bone repair [31]. Bone healing is influenced not only by the defect size, but also depends on the age and strain of the patient and vascularization. In addition, surgical techniques that might destroy the dura mater which (the source of significant osteoprogenitors) is another factor which influence the process of bone reconstruction [33].

Our results on bone regeneration in the control group was similar to the study of Cooper et al. that showed incomplete healing for 2.3 mm bone defects in Sprague-Dawley rats 6 weeks following operation. Also, they demonstrated that the treatment did not improve bone healing after 8 weeks as the defects were filled by soft fibrous tissue suggesting the critical period of restoring bone in this defect is between 4 and 8 weeks in rodents [34]. This is in agreement to our H&E staining observations which also showed the soft tissue in the defect of the control groups (without scaffold) after 4 weeks with a few new bone areas 8 weeks following surgery.

The bone defect was obvious in blank control groups after 4 weeks. This was similar to the study of Ruan et al. that the bone recovery was limited in repair of bone defects in rabbits [35]. However, the bone regeneration increased following 8 weeks of implantation, there was no bridges of bone to create the unified bone joint to be recognized by passing the time. This was in accordance to the study of Liu et al. that implanted composite of adipose-derived stem cells seeded with heterogeneous deproteinized bone to repair the bone defects and lead to non-union bone healing following 8 weeks post-surgery [36].

Previous studies reported that the optimum scaffold pore size for hard tissue regeneration is between 200 and 500 μm [37]. Some proposed a pore size in the range of 75–250 μm [38] or the range from 100 to 500 μm for bone ingrowth [39, 40]. Our results indicated that a pore size of 500 μm showed the highest amount of newly formed bone compared to 250 μm scaffolds. Among the other structures, the heterogeneous grad.250top showed significantly greater new bone formation. However, it has been suggested that an increase in the pore size from 500 μm to a millimeter promoted a loss of mechanical strength of the scaffold, although inducing larger amounts of bone formation due to the flow transport of oxygen and nutrients in the larger pore size [41].

Although, the weak mechanical properties of the larger pore sizes of 500 and 750 μm scaffolds were similar to our previous findings and might not be suitable in terms of mimicking the mechanical strength of bone tissue, the tensile properties of the gradient porous scaffolds including both small and large pore sizes (250, 500 and 750 μm) increased. The smaller pore of 250 μm in the gradient architecture could compensate for the poor mechanical properties of the larger pore sizes of 500 and 750 μm [10]. Also, in our current study we realized that a gradient porosity promoted the highest bone volume compared to other structures. This was in accordance to the study of Wang et al. who implanted a porosity-graded calcium polyphosphate scaffold and showed higher osteoblast colonization in the macropores in contrast to the micropores of the gradient scaffold [42].

In the micro-CT and histologic evaluations, more bone growth and penetration into the pores was attributed to the higher permeability of the gradient and 500 μm scaffolds which is able to increase the delivery of nutrients and O_2_ as well as removing waste products from the larger pores of the construct [43]. But the offset and 250 μm scaffolds with narrower pore sizes were more prone to blockage with soft tissue as the limitation of oxygen and nutrients diffusion that leading to inhibition of bone cell migration and bone growth [44]. In grad 250.top scaffolds that has the smaller pore size (250 μm) on the outside region of scaffold, i.e. near the scalp and the larger pores (750 μm) on inside towards the dura mater, more pronounced newly formed bone was observed in contrast to the grad.750.top where the direction of the gradient pores are reversed. This was probably associated with the dura mater which can provide further blood supply and nutrients into the larger pore (750 μm) of grad.250.top scaffolds. However, in the bottom regions of grad.750top scaffold which contains small pores of 250 μm, few newbone formations were founded due to insufficient blood and nutrient circulation in the narrow pores of the scaffold which is adjacent to the dura mater [45].

The expression of major markers of bone differentiation i.e. Opn, Col I and Ocn are linked with the maturation of osteoblasts and bone development [46]. The degree of Ocn expression was significantly more in grad.250.top scaffolds which promoted more bone formation and mineralization in this scaffold structure. This is consistent with previous studies which reported more mineral accumulation to recover bone defect through Ocn expression [47]. Similar results were found by Tera et al. who observed intense staining of Ocn on the newly formed bone 45–60 days after polytetrafluoroethylene membrane implantation in the mandible of rats [48]. The intense staining of Opn in all scaffolds after 8 weeks showed that normal mineralization of new bone was regulated by Opn as has been previously demonstrated.

Bone remodeling increases the activity of mature osteoclastic resorption via the modulation of Ca^2+^ and Mg^2+^ ion homeostasis releasing high levels of collagen into the mineralized matrix bone [49]. This is in accordance with our findings where intense staining of Collagen was seen in all the scaffolds 8 weeks post-implantation. Two studies have stated the expression of Collagen Type I was enhanced in bone ECM mineralization which is a valuable biomarker of bone remodeling/ mineralization starting approximately two weeks after implantation, only after the morphology transition of new bone to the native tissue occurred [50, 51].

Rapid new bone formation is influenced by the mutual promotion of osteogenesis and angiogenesis [52]. Additionally, other findings demonstrated that endothelial cells might stimulate osteogenesis via raising the level of BMP-2 [53]. The present data also showed a high level of BMP-2 expression 8 weeks following implantation which promoted bone formation particularly in the 500 μm scaffold. Furthermore, the high level of Alp expression as well as BMP-2 was observed for this group after 8 weeks. This finding was similar to the study of Nguyen et al. who reported Alp activity was influenced by BMP-2 release [54].

The interactions between endothelial cells and the osteoblasts that promote bone healing also leads to vascular growth initiated through VEGF [55]. VEGF is a common angiogenic growth factor that induces the development of tubular structures through the growth, proliferation and migration of endothelial cells [56]. VEGF is involved in the entire bone healing process, from hematoma formed at a bone crack to the bone remodeling phase. The synergistic role of VEGF and BMP has been illustrated in bone healing and vascularization [57]. Our immunostaining results also detected up-regulation of VEGF and intense staining of Bmp-2 in all the scaffolds 4 and 8 weeks following implantation. The highest staining of VEGF was observed in offset.50.50 scaffolds after 8 weeks compared to other groups. This might be related to hypoxic conditions in the smaller pores of these two scaffold structures and the expression of hypoxia inducible factor-1α which activates in anaerobic situations to promote the upregulation of VEGF as a proangiogenic factor expanding blood vessel ingrowth and consequently increased oxygen tension in these structures that resulted in limited bone formation [58]. This agrees with the study of Wu et al. that demonstrated the stimulation of angiogenesis in hypoxia-mimicking mesoporous bioactive glass scaffolds with osteogenic properties [59]. The intense staining of VEGF in 500 μm and gradient scaffolds was lower than offset.50.50 scaffolds due to adequate large pore size in these constructs enabling sufficient oxygen, facilitating capillary sprouting and revascularization in the defect site, and successfully stimulating the union of bone formation in these implanted scaffolds. Also, VEGF expression which promotes osteoblast differentiation, is low at the begining of osteoblastogenesis but increases in parallel with the expression of Ocn resulting in mineralization during terminal bone differentiation [60] The higher expression of Ocn along with VEGF may cause the rapid new bone formation seen in the grad.250top scaffold as shown in the micro-CT analysis.

The upregulation of CD34 in all the scaffold groups at 4 weeks was an indication of primitive endothelial tube formation and early angiogenic differentiation in all the structures [61] that promotes bone formation. The study of Hertweck et al. also demonstrated that the positive CD34 cells enhanced the bone volume in a co-culture system with human osteoblast cells [62]. In comparison to the other protein markers, the low level of CD105 expression (a mesenchymal stem cell marker) in our scaffold groups corresponded to previous reports [63, 64]. However, the higher expression of CD105 in offset.50.50 and 250 μm scaffolds could be linked with the inhibition of osteogenesis [65]. Although recent studies reported CD 105 lead to mature and stable vessel formation by adhesion between mesenchymal stem cells and endothelial colony forming cells which enhances neovascularization [66–68]. The presence of vWF confirmed angiogenesis in all treatment groups. These results showed strong expression of vWF in the larger pore size of 500 μm scaffolds had great potential of bone formation which was promoted by increasing vascular functions and blood vessel formation. This was in agreement with other works that observed more and faster osteogenesis, angiogenesis and uniform new formed bone distribution in larger pore sizes than those with the smaller pores [69]. The grad.250top scaffold also exhibited more new bone tissue growth throughout the scaffold. This may be due to the gradient porous structure allowing more tissue ingrowth and blood vessels that result in a cooperative angiogenesis mechanism of CD34 and VEGF along with Ocn at week 8 and vWF at week 4 that increased the speed of new bone regeneration through the nutrient and oxygen flow improvement [70] in comparison to smaller pore sizes. This is in accordance with the studies of Gao and Bolander who demonstrated that the Ocn + CD34+ cell population displayed more mature osteoblasts and mineralized matrix tissue in vitro [71, 72] which is due to the paracrine effect of CD34^+^ cells that causes the secretion of VEGF and their synergistic angiogenesis/ osteogenesis signals on bone regeneration [73].

The resorption of alveolar bone tissue in both horizontal and vertical directions occurs rapidly in the first 3–6 months following dentition loss [74]. While considerable progress has been made in bone augmentation of horizontal bone defects, reproducible results in the treatment of vertical defects is still challenging. Promising results were first observed using non-resorbable membranes due to their impenetrability to soft tissue infiltration which clinically,is considered as a limiting factor [75]. This is due to the lack of vascular supply that facilitates better permeation of nutrients, oxygen and bioactive substances that is vital for the regenerating bone tissue [76]. However, using resorbable large pore size membranes resulted in the migration of soft tissue into the membrane, the potential problems of initial clot formation, the occlusive property of overpopulated soft tissue in the defect site [77] and faster degradation of the membrane before the maturation of vascular tissue [78] which all these inhibit the activity and infiltration of bone-forming cells during vertical bone defects healing. In fact, the architecture and the design of the polymeric porous membranes are therefore important factors in any scaffold osteopromotive effect in vivo to prevent an occlusive barrier while providing adequate space for significant bone growth and vascularization.

Previous studies showed the preservation of the osteogenic components was achieved using meshes with 10 μm pores in the defect space [79, 80]. Larger pores (800–1000 *μ*m) in the membrane promoted greater bone regeneration allowing sufficient vascularization of the implanted graft [81]. Therefore, using gradient porous structures hinders soft tissue infiltration by the small pores of the inner layer that lead to filling the defect with mature bone, while allowing the penetration of nutrients and vascular tissue via the large pores of the external layer. Also, the interspace between the two layers allows the integration of the regenerated bone tissue and the overlying soft tissue [82]. In fact, the vertical pore gradient architecture promotes the mature bone regeneration indirectly by acting as a passive barrier for the invasion of soft-tissue.

## Conclusions

The aim of this study was to assess in vivo, the capacity of MEW fabricated scaffolds with similar structure to native bone tissue in terms of porosity and porous structure, to facilitate the production of new osseous tissue. In this study, we successfully implanted the porous MEW PCL scaffolds into a rat calvarial defect and evaluated the effect of gradient architecture on bone formation. Although the expression of angiogenesis and osteogenesis markers contributed to bone repair in all the scaffold groups, the constructs with a larger pore size such as 500 μm and the gradient structures, showed faster repair of critically-sized bone defects with newly formed bone coverage. However, the gradient porous architecture (grad.250top) with the larger pore size exposed to the dura mater resulted in significant enhancement of bone regeneration compare to other pore size scaffolds i.e. 500 μm and grad.750top. In summary, the MEW PCL scaffold with porosity gradient appears to be a suitable candidate to enhance the bone regeneration.

## Data Availability

The datasets used and/or analyzed supporting the conclusions of current study are available and will be presented by the corresponding author on reasonable request.

## Abbreviations

ALP: Alkaline phosphatase
ANOVA: Analysis of variance
Bmp-2: Bone morphogenetic protein-2
BV: Bone volume
CaP: Calcium phosphate
CD105: Endoglin
Col I: Collagen type I
DAB: 3,3-diaminobenzidine
ECM: Extra cellular matrix
H&E: Haematoxylin and eosin
IHC: Immunohistochemistry
MEW: Melt electrowriting
μ-CT: Micro-computed tomography
Ocn: Osteocalcin
Opn: Osteopontin
PCL: Poly(ε-caprolactone)
PFA: Paraformaldehyde
PTFE: Polytetrafluoroethylene
RT: Room temperature
SC: Sub cutaneous
SPSS: Statistical Package for Social Sciences
TV: Total volume
VEGF: Vascular endothelial growth factor
vWF: Von Willebrand factor
